# Reorganization of intrinsic functional connectivity in early-stage Parkinson’s disease patients with probable REM sleep behavior disorder

**DOI:** 10.1038/s41531-023-00617-7

**Published:** 2024-01-03

**Authors:** Xiao-Juan Dan, Yu-Wei Wang, Jun-Yan Sun, Lin-Lin Gao, Xiao Chen, Xue-Ying Yang, Er-He Xu, Jing-Hong Ma, Chao-Gan Yan, Tao Wu, Piu Chan

**Affiliations:** 1 Department of Neurology, Xuanwu Hospital of Capital Medical University, 100053 Beijing, China; 2 Key Laboratory on Neurodegenerative Disorders of Ministry of Education, Key Laboratory on Parkinson’s Disease of Beijing, 100053 Beijing, China; 3 CAS Key Laboratory of Behavioral Science, Institute of Psychology, 100101 Beijing, China; 4 Department of Psychology, University of Chinese Academy of Sciences, 100101 Beijing, China; 5 Center for Movement Disorders, Department of Neurology, Beijing Tiantan Hospital, Capital Medical University, 100070 Beijing, China; 6 Department of Neurobiology, Xuanwu Hospital of Capital Medical University, 100053 Beijing, China; 7 National Clinical Research Center for Geriatric Disorders, 100053 Beijing, China; 8 Beijing Institute for Brain Disorders Parkinson’s Disease Center, Advanced Innovation Center for Human Brain Protection, Capital Medical University, 100069 Beijing, China

**Keywords:** Parkinson's disease, Parkinson's disease

## Abstract

REM sleep behavior disorder (RBD) symptoms in Parkinson’s disease (PD) suggest both a clinically and pathologically malignant subtype. However, whether RBD symptoms are associated with alterations in the organization of whole-brain intrinsic functional networks in PD, especially at early disease stages, remains unclear. Here we use resting-state functional MRI, coupled with graph-theoretical approaches and network-based statistics analyses, and validated with large-scale network analyses, to characterize functional brain networks and their relationship with clinical measures in early PD patients with probable RBD (PD+pRBD), early PD patients without probable RBD (PD-pRBD) and healthy controls. Thirty-six PD+pRBD, 57 PD-pRBD and 71 healthy controls were included in the final analyses. The PD+pRBD group demonstrated decreased global efficiency (t = -2.036, P = 0.0432) compared to PD-pRBD, and decreased network efficiency, as well as comprehensively disrupted nodal efficiency and whole-brain networks (all eight networks, but especially in the sensorimotor, default mode and visual networks) compared to healthy controls. The PD-pRBD group showed decreased nodal degree in right ventral frontal cortex and more affected edges in the frontoparietal and ventral attention networks compared to healthy controls. Furthermore, the assortativity coefficient was negatively correlated with Montreal cognitive assessment scores in the PD+pRBD group (r = -0.365, P = 0.026, d = 0.154). The observation of altered whole-brain functional networks and its correlation with cognitive function in PD+pRBD suggest reorganization of the intrinsic functional connectivity to maintain the brain function in the early stage of the disease. Future longitudinal studies following these alterations along disease progression are warranted.

## Introduction

Rapid eye movement (REM) sleep behavior disorder (RBD) is a parasomnia characterized by loss of the normal muscular atonia during REM sleep with complex motor behaviors accompanying vivid dreaming^1^. As a clinical harbinger of subsequent α-synucleinopathies^2^, RBD symptom is often a common nonmotor comorbidity found in about half of patients with Parkinson’s disease (PD)^3,4^. Empirically, it has been observed that PD patients with RBD often show more severe cognitive and/or motor deficits than those without RBD, even at early stages of the disease^5–8^. In addition, longitudinal data suggest that RBD is associated with a more rapid motor progression and cognitive decline in patients with PD^9,10^. Finally, the presence of RBD in PD is linked to an increased risk of dementia and prone to have a malignant prognosis^11–14^.

The underlying pathophysiology for RBD in PD remains incompletely understood, especially in early disease stages. A postmortem study revealed more diffuse and severe deposition of α-synuclein pathology in PD patients with RBD^15^. Neuroimaging techniques, especially resting-state functional MRI (rs-fMRI), may also provide insight into the relations between RBD and PD. Previous fMRI studies supported that the presence of RBD in PD patients is associated with functional alterations in several brain regions^16–19^. In addition, rather than only based on isolated regions, recent whole-brain network analyses allow investigations into alterations in segregation and integrated information processing^20,21^. To our knowledge, only two studies have explored whole-brain functional network alterations in PD patients with probable RBD (PD+pRBD) using complex network analysis so far. Coupled with whole-brain network-based statistics (NBS) and graph-theoretical approaches (GTA) analyses, Oltra et al. reported that PD+pRBD showed reduced brain functional connectivity (FC) compared to healthy controls and disrupted posterior functional connectivity as well as increased normalized characteristic path length compared to patients without RBD (PD-pRBD)^22^. However, the other previous research performed in PD+pRBD showed inconsistent results and did not find difference in global graph metrics between groups^23^. Moreover, both the previous studies investigated the pRBD in PD patients with an aggregation of different stages. Up to now, no study has fully explored the possible alterations in intrinsic functional brain architecture in PD+pRBD with a focus on the early-stage of the disease.

To assess if the presence of RBD symptoms affects the organization of whole-brain intrinsic functional networks in early PD and whether a unique and specific brain network FC pattern exists in this patient subgroup, here we apply GTA and NBS analyses, combined with large-scale network analyses as validation, to examine functional brain networks and their relations with clinical features in early PD+pRBD. As a diffuse/malignant PD subtype, in addition to clinical and pathological observations^6,12–15^, PD+pRBD subjects have more severe neuropsychological and functional brain-imaging alterations already at the time of PD diagnosis^24^. Moreover, de novo PD patients with pRBD also have disrupted topological organization of white matter in the whole brain^24^. Additionally, some altered imaging metrics in PD+pRBD have associated with clinical features, especially the cognitive function^17,18,22^. A recent study reported that even in drug-naïve and cognitively unimpaired PD+pRBD, resting-state FC changes within neurocognitive networks were already detectable^25^. Thus, in this study, we hypothesized that early PD+pRBD would show decreased efficiency in brain networks and extensively disrupted brain FC compared to PD-pRBD and/or healthy controls, and these imaging alterations may be correlated with the cognitive function in PD+pRBD.

## Results

### Demographic and clinical characteristics

In total, 93 PD patients (36 PD+pRBD and 57 PD-pRBD) and 71 healthy controls were included in the final sample after implementing MRI quality control measures (Supplementary Fig. 1). PD patients and healthy controls were matched with respect to age, sex and educational level. PD+pRBD and PD-pRBD had comparable age at disease onset, disease duration, Hoehn and Yahr (H&Y) stages, Movement Disorder Society Unified Parkinson’s Disease Rating Scale (MDS-UPDRS) part II and part III scores. The PD+pRBD group had significantly higher RBD questionnaire-Hong Kong version (RBDQ-HK) scores, 17-item Hamilton Rating Scale for Depression (HAMD-17) scores, Pittsburgh Sleep Quality Index (PSQI) scores, MDS-UPDRS total and part I scores, and lower Montreal cognitive assessment (MoCA) scores compared to PD-pRBD, and higher RBDQ-HK, HAMD-17, PSQI and lower MoCA, Brief Smell Identification Test (B-SIT) scores compared to healthy controls. Higher HAMD-17 scores and lower B-SIT scores were found in the PD-pRBD group compared to healthy controls (Table 1). Based on bed-partner interviews, improvements in movement and speech during RBD episodes, relative to awake periods, were observed for PD+pRBD (see Supplementary Information).

**Table 1:** Demographics and clinical characteristics of PD + pRBD, PD-pRBD and HC groups.

	PD+pRBD (*n* = 36)	PD-pRBD (*n* = 57)	HC (*n* = 71)	*P*-value
Age, mean ± SD, years^a^	61.2 ± 6.5	58.4 ± 8.8	60.5 ± 8.3	0.202
Sex, male, *n* (%)^b^	19 (52.8)	26 (45.6)	32 (45.1)	0.729
Education level, median ± IQR, years^c^	11.0 ± 5.0	9.0 ± 6.0	12.0 ± 6.0	0.182
Age at onset, mean ± SD, years^d^	57.5 ± 7.3	55.5 ± 9.1	NA	0.329
Disease duration, median ± IQR, years^e^	3.0 ± 3.0	2.0 ± 2.5	NA	0.161
MDS-UPDSR, mean ± SD^d^	43.1 ± 16.7	35.9 ± 15.9	NA	**0.039**^f^
MDS-UPDRS part I, median ± IQR^e^	9.0 ± 6.0	5.0 ± 5.0	NA	**<0.001**^f^
MDS-UPDRS part II, median ± IQR^e^	9.0 ± 7.7	7.0 ± 8.0	NA	0.321
MDS-UPDRS part III, median ± IQR^e^	24.5 ± 18	23.0 ± 15.5	NA	0.220
H&Y stage, *n*, 1/2/2.5^b^	7/23/1	23/26/2	NA	0.067
MoCA, median ± IQR^c^	24.0 ± 3.7	26.0 ± 4.0	26.0 ± 4.0	**<0.001**^f,g^
HAMD-17, median ± IQR^c^	8.0 ± 7.7	4.0 ± 4.0	1.0 ± 4.0	**<0.001**^f,g,h^
RBDQ-HK, median ± IQR^c^	28.5 ± 14.5	3.5 ± 8.7	7.0 ± 9.0	**<0.001**^f,g^
ESS, median ± IQR^c^	4.0 ± 4.0	3.0 ± 4.0	4.0 ± 3.0	0.464
PSQI, median ± IQR^c^	8.0 ± 6.0	3.0 ± 4.0	4.0 ± 5.0	**0.008**^f,g^
AS, median ± IQR^c^	10.0 ± 17.0	6.0 ± 14.0	4.0 ± 10.0	0.066
B-SIT, median ± IQR^c^	7.0 ± 4.0	8.0 ± 3.5	10.0 ± 3.0	**<0.001**^g,h^

*RBD* Rapid Eye Movement Sleep Behavior Disorder, *pRBD* probable RBD, *PD* + *pRBD* PD with probable RBD, *PD-pRBD* PD without probable RBD, *HC* healthy control, *MDS-UPDRS* Movement Disorder Society Unified Parkinson’s Disease Rating Scale, *MDS-UPDRS part I* Non-motor Aspects of Experiences of Daily Living section of MDS-UPDRS, *MDS-UPDRS part II* Motor Aspects of Experiences of Daily Living section of MDS-UPDRS, *MDS-UPDRS part III* Motor Examination section of MDS-UPDRS, *H&Y* Hohen & Yahr scale, *MoCA* Montreal Cognitive Assessment, *HAMD* Hamilton Depression scale, *RBDQ-HK*, RBD Questionnaire-Hong Kong version, *ESS* Epworth Sleepiness Scale, *PSQI* Pittsburgh Sleep Quality Index, *AS* Apathy Scale, *B-SIT* Brief Smell Identification Test, *SD* standard deviation.

*P* values with statistically significant differences are highlighted in bold text.

^a^Analysis of variance (ANOVA) followed by post hoc test corrected by Bonferroni was used.

^b^The *Χ*^*2*^ was used.

^c^Kruskal-Wallis H-test followed by post hoc test corrected by Bonferroni was used.

^d^Variables with mean ± SD compared via independent *t*-test.

^e^Variables with median ± IQR compared via Mann-Whitney U test.

^f^Comparison between PD+pRBD group and PD-pRBD group.

^g^Comparison between PD+pRBD group and healthy control group.

^h^Comparison between PD-pRBD group and healthy control group.

### Whole-brain functional topological properties

**Fig. 1:**
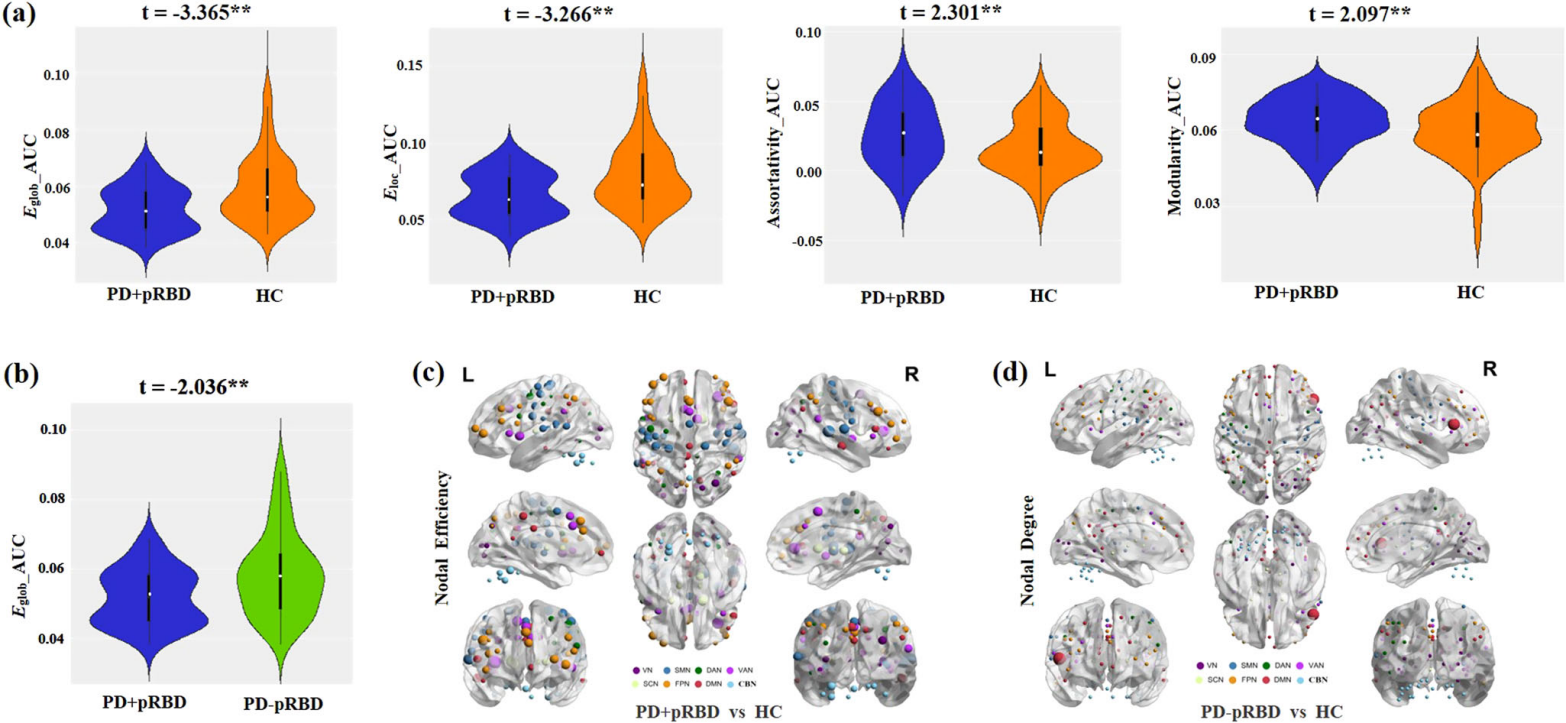
Group differences in network topological properties between PD + pRBD, PD-pRBD and healthy controls (HC). **a** Violin plots illustrating the area under the curve (AUC) parameters of the global efficiency (E_glob_), local efficiency (E_loc_), assortativity coefficient and modularity for PD+pRBD and HC. White dots indicate the sign for the medians. The thicker black lines represent the quartile range, while the thinner black lines represent 95% confidence interval in the violins. **b** Violin plots illustrating the AUC parameters of E_glob_ for PD+pRBD and PD-pRBD. The t values are the statistics for these comparisons in general linear model analyses. **c** Group differences between PD+pRBD and HC in efficiency at the nodal level. The spheres denote significant differences after FDR correction and the size of the spheres dependent on the rank of the P values (i.e, the smaller the P value, the larger the size of the spheres). The color of the nodes reflects the networks of the brain. **d** Group differences between PD-pRBD and HC in nodal degree. RBD Rapid Eye Movement Sleep Behavior Disorder, pRBD probable RBD, PD + pRBD PD with probable RBD, PD-pRBD PD without probable RBD, VN visual network, SMN somatosensory network, DAN dorsal attention network, VAN ventral attention network, SCN subcortical network, FPN frontoparietal network, DMN default mode network, CBN cerebellar network. ** p < 0.05 (p value for general linear model analyses, two-tailed).

Alteration of whole-brain network topologies in PD+pRBD was revealed in groups comparison using age, sex, educational level, and mean frame-wise displacement (FD) as covariates. Network efficiency values, E_glob_ (t = -3.365, P = 0.0006, d = 0.112) and E_loc_ (t = -3.266, P = 0.0016, d = 0.106) were significantly decreased, while assortativity coefficient (t = 2.301, P = 0.022, d = 0.052) and modularity value (t = 2.097, P = 0.036, d = 0.044) were significantly increased in PD+pRBD compared to healthy controls (Fig. 1a). When compared to PD-pRBD, PD+pRBD had significantly decreased global efficiency value (t = -2.036, P = 0.0432, d = 0.0477) **(**Fig. 1b). The differences of global brain properties between PD-RBD and healthy controls did not reach statistical significance.

For regional nodal features, after false discovery rate (FDR) correction, the PD+pRBD group had decreased nodal efficiency in several brain areas, including the subcortical network (SCN) (left basal ganglia, left thalamus), somatosensory-motor network (SMN) (bilateral parietal lobe, bilateral temporal lobe, bilateral precentral gyrus, left SMA), frontoparietal network (FPN) (bilateral dorsal lateral prefrontal cortex, bilateral ventral prefrontal cortex, left anterior cingulate cortex, left occipital lobe), dorsal attention network (DAN) (left occipital lobe, left precentral gyrus, right intraparietal sulcus), cerebellar network (CBN) (bilateral cerebellar), visual network (VN) (bilateral post occipital lobe), ventral attention network (VAN) (bilateral parietal lobe, right middle insula), and default mode network (DMN) (right post cingulate, bilateral precuneus) and increased nodal efficiency in VN (left occipital lobe) compared to healthy controls (Fig. 1c). Compared to healthy controls, PD-pRBD had a decreased nodal degree in DMN (right ventral frontal cortex) (Fig. 1d). The nodal features in other comparisons did not survive after FDR correction.

### Edge-based functional connectivity

**Fig. 2:**
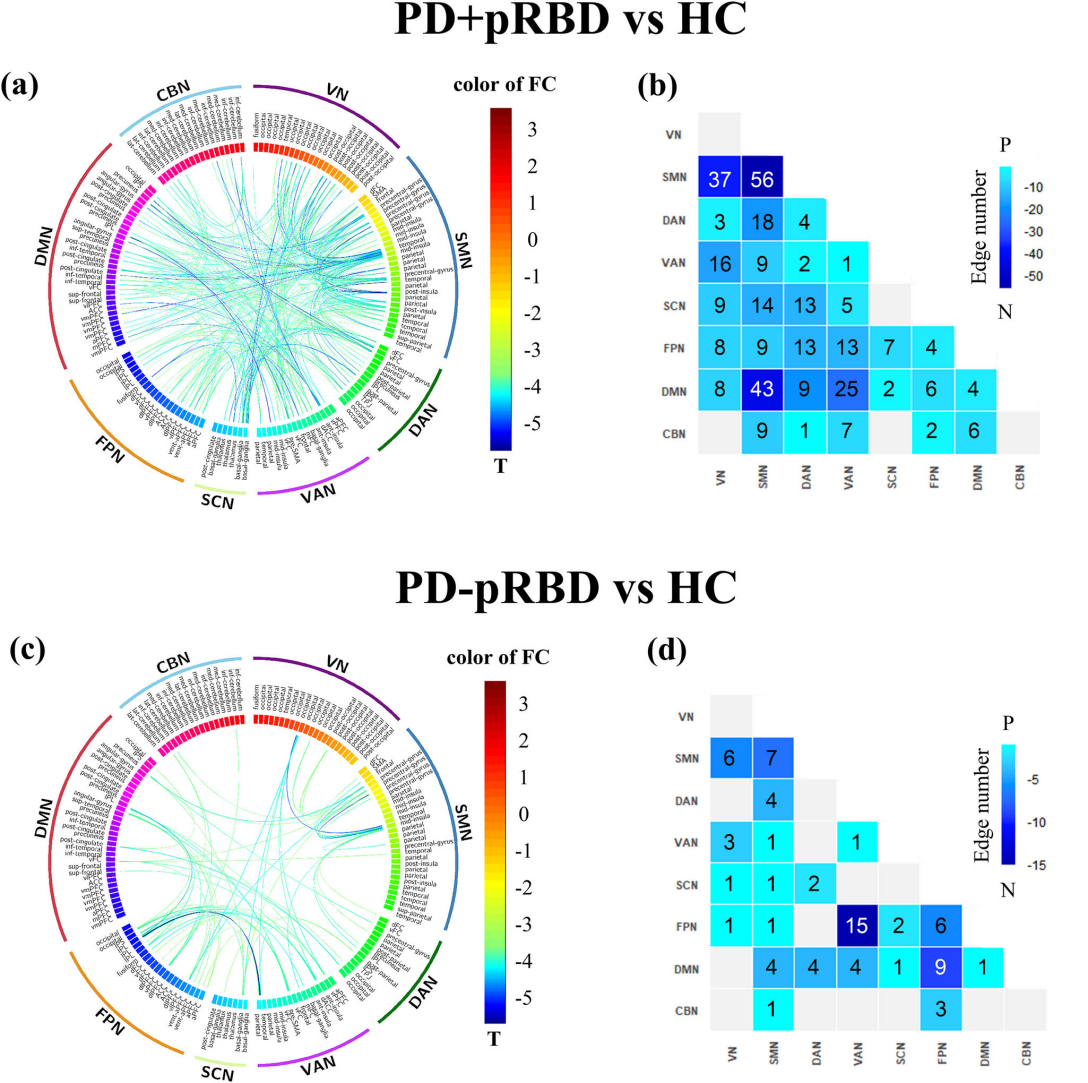
Connectograms of significant within-and between-network connections in Network-Based Statistics (NBS) analyses between patient-control comparisons. The comparison between PD+pRBD and HC was plotted in the left circular graph (**a**) or in the right heatmap (**b**). For the circular graph, the line color indicates differences in significant t values showed by the color map. For the heatmap, the number indicates differences in significant edges for each pair of networks showed by the color map. The comparison between PD-pRBD and HC was plotted in the left circular graph (**c**) or in the right heatmap (**d**). RBD Rapid Eye Movement Sleep Behavior Disorder, pRBD probable RBD, PD + pRBD PD with probable RBD, PD-pRBD PD without probable RBD, VN visual network, SMN somatosensory network, DAN dorsal attention network, VAN ventral attention network, SCN subcortical network, FPN frontoparietal network, DMN default mode network, CBN cerebellar network.

The maps of brain edge-based FC matrices of PD+pRBD, PD-pRBD and healthy controls are shown in Supplementary Fig. 2. An NBS analysis of PD+pRBD versus healthy controls revealed a significant cluster consisting of 133 ROIs and 376 edges with decreased FC in PD+pRBD (Fig. 2a). More affected edges were connected to ROIs in SMN, VN, DMN, and fewer in the CBN (Fig. 2b, Supplementary Table 1). The most significantly decreased edge involved ROIs within SMA (T = -5.157, P = 0.0002, d = 0.263). In contrast, an NBS analysis of PD-pRBD versus healthy controls revealed a significant cluster of 52 ROIs and 78 edges with decreased FC in PD-pRBD (Fig. 2c). More affected edges were involved ROIs in FPN and VAN, and fewer in the SCN and CBN (Fig. 2d, Supplementary Table 2). The most significant decreased edge was also connected to ROIs in FPN and VAN (T = -5.617, P = 0.0002, d = 0.259). However, PD+pRBD versus PD-pRBD did not reach a statistically significant difference in the NBS analysis.

### Correlations between network metrics and clinical measures

**Fig. 3:**
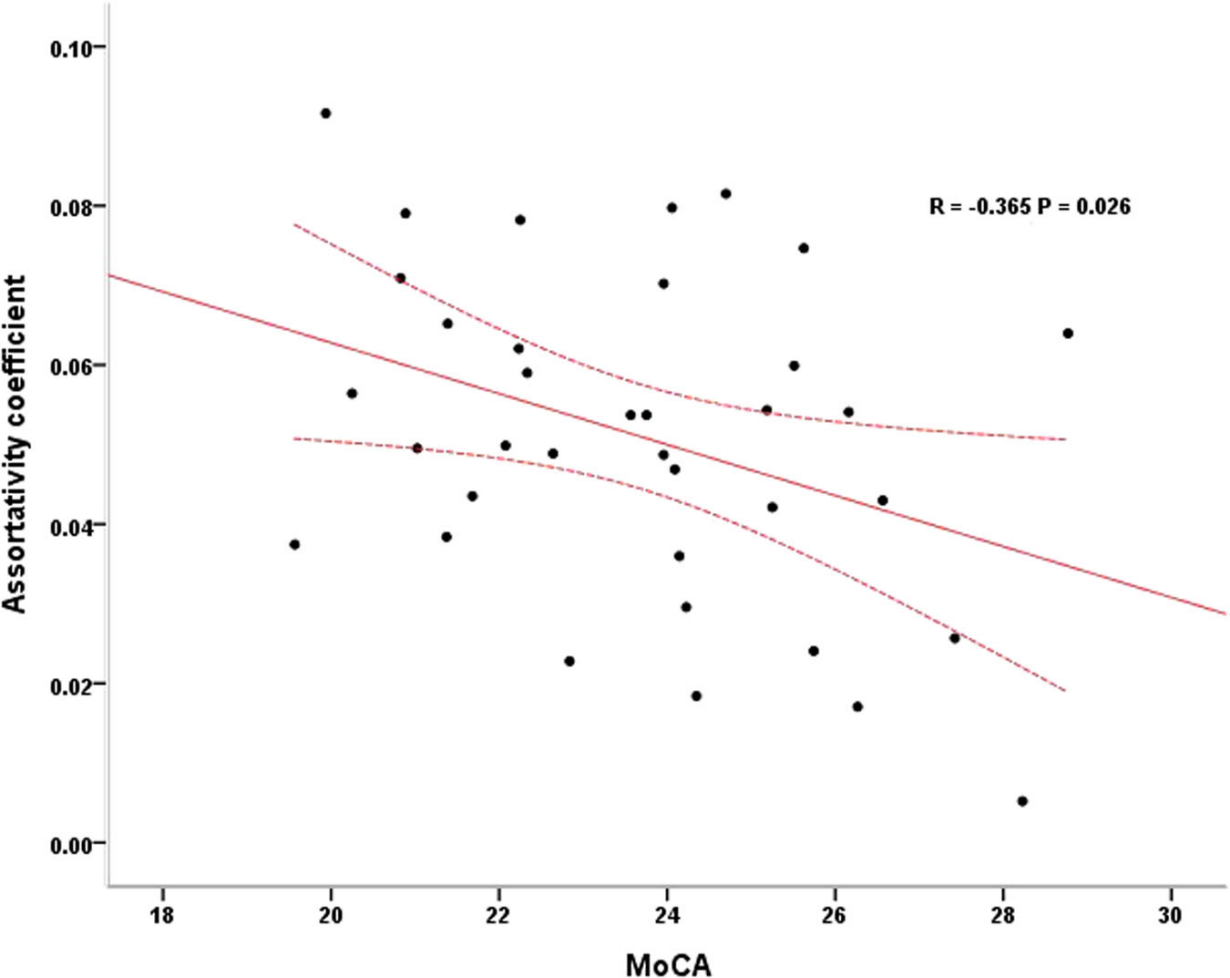
The partial correlation between assortativity coefficient and MoCA scores in PD + pRBD. The assortativity coefficient was negatively correlated with MoCA scores in PD+pRBD after controlling for age, sex, educational level and mean frame-wise displacement. *MoCA* Montreal Cognitive Assessment, *PD* + *pRBD* Parkinson’s disease with probable rapid eye movement sleep behavior disorder. The area between the dotted lines represents 95% confidence interval.

The assortativity coefficient was significantly negatively correlated with MoCA scores in PD+pRBD after controlling for age, sex, educational level and mean FD (r = -0.365, P = 0.026, d = 0.154, Fig.3).

No significant correlations between the edge-based FC of significant clusters or the other network topological features in PD+pRBD and the clinical features (i.e., MoCA score, HAMD score, UPDRS motor score, H&Y stage, disease duration) were observed after FDR correction, when controlling for age, sex, educational level and mean FD.

No significant correlations between these network metrics and clinical measures were found in healthy controls and PD-pRBD groups.

### Validation analyses

Analysis of two confirmatory metrics (path length and clustering coefficient) confirmed our primary topological findings. Specifically, PD+pRBD showed significantly higher path length values (t = 3.511, P = 0.0006, d = 0.122) and lower clustering coefficient values (t = -3.012, P = 0.0028, d = 0.090) than healthy controls.

**Fig. 4:**
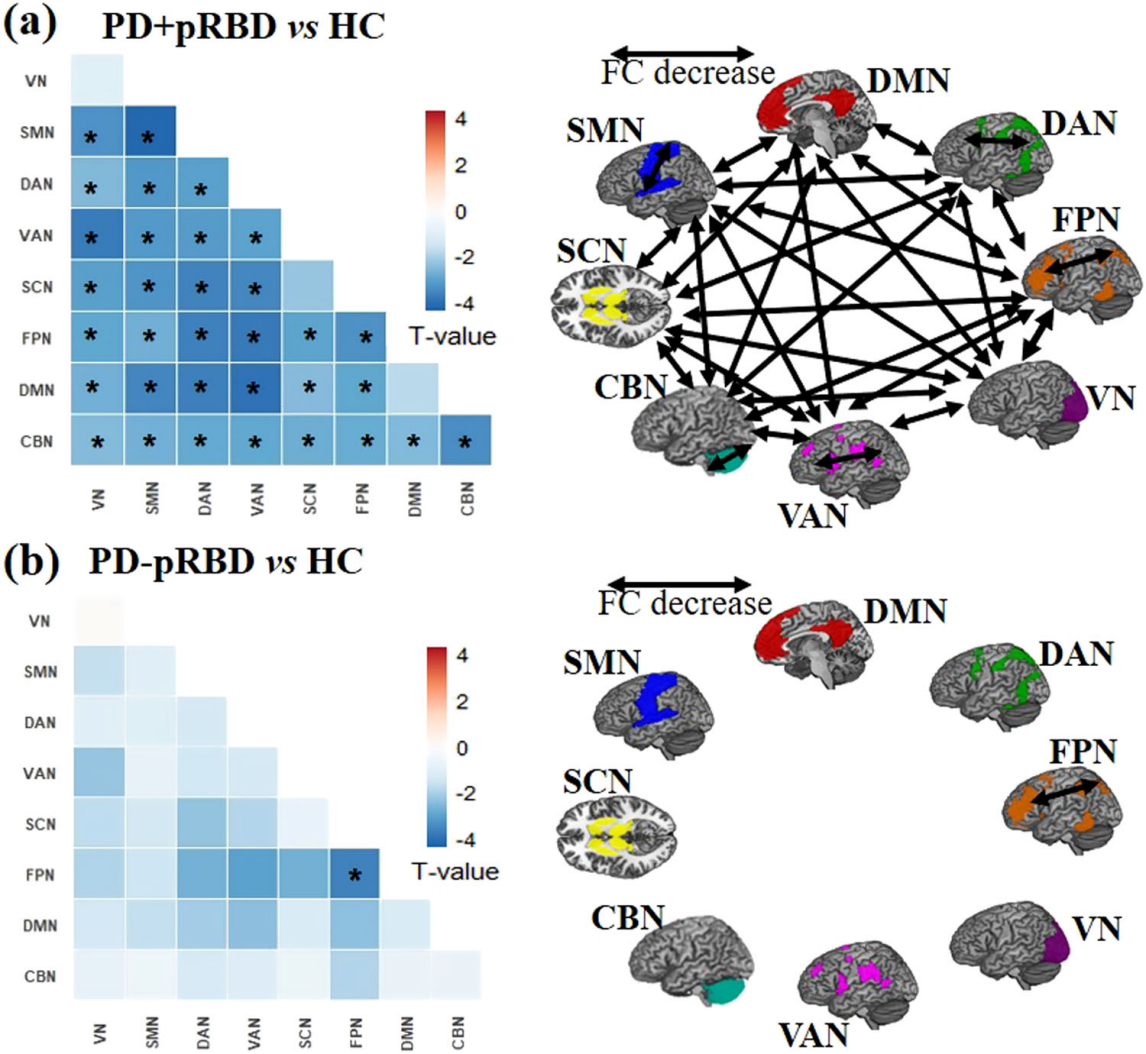
The large-scale within- and between-network FC comparisons between PD + pRBD, PD-pRBD and healthy controls (HC). **a** The upper-left heatmap shows the T values of two sample T tests on large-scale network FC comparison between PD+pRBD and HC. The schematic diagram on the upper-right panel shows the network connections with significant FC decrease for the eight networks between PD+pRBD and HC. **b** The lower-left heatmap shows the T values of two sample T tests on large-scale network FC comparison between PD-pRBD and HC. The schematic diagram on the lower-right panel shows the network connections with significant FC decrease for the eight networks between PD-pRBD and HC. FC functional connectivity, RBD Rapid Eye Movement Sleep Behavior Disorder, pRBD probable RBD, PD + pRBD PD with probable RBD, PD-pRBD PD without probable RBD, HC healthy controls VN visual network, SMN somatosensory network, DAN dorsal attention network, VAN ventral attention network, SCN subcortical network, FPN frontoparietal network, DMN default mode network, CBN cerebellar network. * Significant FDR-corrected p < 0.05 (two-tailed) among eight within network and 28 between-network connections.

In addition to the NBS analyses alluded to above, we also validated the edge-based FC results by analyzing large-scale within- and between-network FC. We found extensively decreased within- and between-network FC in PD+pRBD as well as decreased within-network FC of FPN in PD-pRBD compared to healthy controls in a large-scale network analysis (Fig.4 and Supplementary Table 3, 4). A further validation analysis using an alternative brain parcellation approach essentially confirmed our original main edge-based FC findings (see Supplementary Information).

Structurally, no significant clusters survived in voxel-based morphometry (VBM) analysis between group-contrasts among PD+pRBD, PD-pRBD and healthy controls after FDR correction. In addition, after further controlling for MoCA scores, the PD+pRBD group showed a significant cluster consisting of 115 ROIs and 244 edges compared to healthy controls in the NBS analysis.

Finally, we examined whole-brain topological properties and edge-based FC (including NBS and large-scale network analyses) between pooled PD patients and healthy controls. Pooled PD patients showed disrupted topological features (E_glob,_ t = -2.643, P = 0.01, d = 0.044; E_loc,_ t = -2.574, P = 0.01, d = 0.042) and extensively altered FC (Supplementary Fig. 3 and Fig. 4, Supplementary Table 5) compared to healthy controls (see Supplementary Information).

## Discussion

The present study validates our hypothesis and has three major findings:

1. In early PD patients, the presence of RBD was associated with significantly decreased global efficiency. The PD+pRBD exhibited significantly altered global and local brain topological properties compared to healthy controls. In contrast, the PD patients without RBD only had decreased nodal degree in the right ventral frontal cortex compared to healthy controls.
2. Compared to healthy controls, edge-based FC analyses demonstrated extensively disrupted brain networks in PD subjects, with the posterior brain (e.g., SMN, VN, DMN) being more affected in the PD+pRBD group, and the FPN and VAN being more affected in the PD-pRBD group.
3. In the PD+pRBD group, the assortativity coefficient was negatively correlated with MoCA scores. The observation of altered whole-brain functional networks and its correlation with cognitive function in PD+pRBD suggest reorganization of the intrinsic functional connectivity to maintain the brain function at the early stage of this disorder.

Our first major finding is that there are distinct functional brain topological properties in PD patients with and without pRBD, even in the early stage of the disease. In the early PD patients, we further confirmed the findings of Oltra et al., which showed decreased global network efficiency in PD+pRBD compared to PD-pRBD^22^.The other previous study did not find any alteration in global network measures in PD+pRBD may likely be due to the difference of sample distribution or the use of an anatomic labeling atlas rather than a functional atlas used for brain parcellation^23,26^. Besides, we also found distinct functional brain topological patterns between PD+pRBD and PD-pRBD groups compared to healthy controls. Only decreased local nodal degree in the right ventral frontal cortex was found in PD-pRBD, while with the presence of RBD, we evidenced extensively altered nodal efficiency, decreased global and local efficiency, and increased assortativity and modularity in PD patients. These results indicate the decreased efficiency of global information transfer as well as altered internodal organization or disrupted integration and separation of the whole brain in PD+pRBD^27,28^, but the preservation of topological network architecture for PD-pRBD, which might reflect the different underlying pathophysiological changes between early PD patients with and without RBD. Previous studies exploring altered brain topological properties in early PD patients showed inconsistent results^29,30^. While stratified by RBD, in this relatively large sample size of functional MRI studies in PD patients with RBD to date^16–19,22,23,31,32^ (Supplementary Table 6), we demonstrated that disrupted functional brain topological organization was mainly involved in the PD+pRBD subtype at the early stage of the disease.

Our second major finding was the implications of RBD on brain FC in early PD. In line with previous functional neuroimaging studies^22,32^, we confirmed severely altered brain networks in PD+pRBD. As expected, remarkably disrupted sensorimotor and subcortical networks were observed in PD+pRBD. Furthermore, we showed that altered FC was more prominent in the posterior part of the brain in PD+pRBD. This is consistent with previous FC studies in polysomnography (PSG)-confirmed patients with idiopathic RBD (iRBD)^33^ and in PSG-confirmed PD-RBD^18^. The latter study also showed lower grey matter volume in these PD patients. Moreover, hypometabolism in posterior cortical regions in patients with de novo PD and pRBD has also been demonstrated in a positron emission tomography (PET) study^34^. On the other hand, after removing the impact of RBD, PD-pRBD mainly showed altered edges in FPN and VAN networks compared to healthy controls, suggesting that the presence of RBD is more associated with changes in the posterior parts of the brain in early PD patients.

A decrease in FC in FPN and VAN networks is usually associated with worsening executive performance and lower attention processing^35,36^, possibly due to the dopaminergic denervation in PD. With the presence of RBD, although FPN and VAN networks were also affected, the FC changes were most prominent in the posterior parts of the brain, including the VN, SMN and DMN, and the affected nodal were more extensive, accompanying with increased nodal efficiency in VN. The presence of RBD symptoms was associated with cholinergic system denervation in PD^37^. Thus, the exacerbation of both dopaminergic and cholinergic denervation could be the joint sources of the extensive and severe disruption of the brain networks we observed in PD+pRBD. The cholinergic system is closely associated with the posterior cortical cognitive subtype (i.e., temporal, parietal, occipital cortices) of PD dementia, according to the “dual syndrome hypothesis”^38,39^. Therefore, it is reasonable to speculate that altered posterior brain function involving the cholinergic system may be a common pathophysiological mechanism for both RBD and cognitive impairment/dementia in PD. Future studies aimed to explore the relationships between the presence of RBD and the “dual syndrome hypothesis” in PD may help clarify the relations between PD dementia and RBD. Recent studies investigating the structural and functional MRI changes associated with PD dementia demonstrated more abundant and extensive white matter alterations and increased intra-network FC within the basal ganglia network in PD patients with the posterior cortical cognitive deficits subtype^40,41^, but the relationship with RBD was not explored.

In the current study, we found a negative correlation between assortativity coefficient and the MoCA scores in PD+pRBD. The assortativity coefficient measures the resilience of the brain networks^28^. The higher assortativity coefficient indicates the networks percolate more easily and are also more robust to vertex removal^28,42^. In the context of extensively disrupted FC and significantly decreased efficient transfer of information of the whole-brain networks, an incremented assortativity coefficient in early PD+pRBD may suggest the reorganization of intrinsic functional connectivity of the whole-brain to compensate the decline of brain function at this stage. Here, the weak but significant negative correlation between assortativity coefficient and cognitive function in PD+pRBD might be a potential compensatory mechanism of cognitive impairment in PD + RBD at the early disease stage before any significant brain atrophy becomes apparent. However, as no other information regarding neuropsychological performance is available in this study and the correlation effect size is limited, more explorations concerning the compensatory explanation need to be conducted in the future. Using GTA and NBS, two previous studies have explored the relationship among RBD, altered imaging metrics and cognitive function. An internodal FC strength positively correlated with lower cognitive performance in iRBD, and no significant correlations between global topological metrics and cognitive function were found^33^. While in a group of pooled PD patients with all disease stages, a significant correlation between the mean functional connectivity strength and a visuoperceptual task, as well as significant correlations between the normalized characteristic path length and measures of mental processing speed and verbal learning were reported in PD+pRBD^22^. Future longitudinal studies with iRBD cohort as well as PD patients with and without RBD cohorts would be required to further understand the pathophysiology and the evolution of brain function with disease progression.

The present study has some limitations that need to be addressed. Firstly, to focus on the early PD and minimize the effect of medication, we restricted our patients with H&Y < 3 and scanned them in the “OFF” medication phase. Thus, the results cannot be generalized to all situations. Secondly, in focusing on the presence of RBD on the brain FC pattern and its correlation with the clinical manifestations, we assessed global cognitive function rather than specific cognitive domains. Studies investigating the relations between specific cognitive domains and brain FC in early disease stages could help to further clarify the impact of RBD on the brain FC and the precise cognition function in this disease. Thirdly, based on purely practical considerations, we only collected rs-fMRI data for PD+pRBD in an awake state rather than during an RBD episode. Previous studies using ictal single photon emission tomography in an RBD episode compared to wakefulness have suggested that the transient improvement of motor function during RBD episodes in PD may be attributable to primary motor cortex activity activating without the inhibitory role of the basal ganglia^43–45^. A future study using fMRI in PD patients with RBD across the sleep-wake cycle, if indeed practical, would assist in our understanding of RBD in patients with PD. Fourthly, the presence of RBD was assessed by a questionnaire rather than confirmed via PSG. We assessed the current RBD symptoms around the time of fMRI scanning but did not stratify the presence of RBD before or after the onset of PD and the duration of RBD symptoms. PD subjects without current obvious RBD symptoms but with RBD history were excluded from this study, although RBD is a robust and stable marker of early PD^46,47^. Finally, the effect sizes of the reported results in this study are limited, probably due to the insufficient sample size. Future long-term prospective follow-up studies with larger sample size, including PSG-confirmed iRBD, PD with RBD and PD without RBD cohorts with precise and comprehensive clinical examination and neuroimaging assessment would be needed to reveal how the evolution of RBD is associated with altered brain function in PD.

In conclusion, this rs-fMRI study investigated altered brain intrinsic functional network profiles in early-stage PD with and without the presence of RBD. PD+pRBD showed decreased brain efficiency and more severely and extensively disrupted brain networks, especially in the posterior parts of the brain compared to healthy controls, and decreased global efficiency compared to PD-pRBD. In addition, the assortativity coefficient was negatively correlated with MoCA scores in PD+pRBD. Our findings provide the whole-brain intrinsic functional network signature for early PD with RBD and shed new light on the underlying pathophysiology for the idea that the presence of RBD in PD indicates a malignant subtype of the disease, which opens further avenues for precise individual interventions in PD.

## Methods

### Participants and clinical assessment

A total of 203 subjects, including 119 patients with PD and 84 matched healthy controls, participated in this study. Patients with PD were recruited from the Parkinson and Movement Disorder Center at the Xuanwu Hospital of Capital Medical University in Beijing. Clinically established diagnoses of idiopathic PD were made based on the Movement Disorder Society (MDS) clinical diagnostic criteria^48^. Patients with a positive family history of PD, secondary parkinsonism, or other forms of atypical parkinsonism were excluded. Age and sex-matched healthy controls were recruited from the Beijing Longitudinal Study on Aging community cohort. None of the elderly control subjects had a history of neurological/psychiatric disorders, nor were they taking any psychoactive medications. All subjects were right-handed and of Chinese Han ethnicity. This study was conducted in accordance with the Declaration of Helsinki and approved by the Ethic Review Committee of the Xuanwu Hospital of Capital Medical University. All participants gave written informed consent before inclusion in this study.

For each participant, demographic information, including date of birth, sex and educational level, and a comprehensive set of motor and non-motor symptoms were assessed. The evaluation of the MDS-UPDRS and H&Y stage was conducted by two movement disorders specialists. To test patients in the early-stage of the disease, only clinically established PD cases with H&Y stage < 3 were included in this study. Mood was assessed using the HAMD-17, and global cognitive functioning was assessed using the MoCA, Beijing version. Participants with moderate to severe dementia (score>1 on the Clinical Dementia Rating Scale) and meeting the Movement Disorders criteria for PD dementia were excluded. We further excluded participants with lower MoCA to avoid the potential outliers (all participants: MoCA ≥20, healthy controls: MoCA ≥20 and mini-mental state examination ≥26). Apathy symptom severity was evaluated with Apathy Scale (AS), and olfactory function was evaluated with the B-SIT^49^.

Sleep disturbance was evaluated by the Epworth sleepiness scale (ESS), PSQI and the validated RBDQ-HK. The RBDQ-HK is composed of 13 questions related to RBD clinical features. Both the overall questionnaire and its subscale of RBDQ-HK(Q6-Q12) have been validated as having reasonable specificity and sensitivity for detecting RBD^50,51^. Based on the RBDQ-HK scores, patients with PD were categorized as PD patients with probable RBD (PD+pRBD, RBDQ-HK score ≥ 19 and subscale ≥ 8) group and PD patients without probable RBD (PD-pRBD, RBDQ-HK score < 19 and subscale < 8) group. PD patients with only RBDQ-HK score ≥ 19 or subscale≥ 8 were not included in this study. To avoid the potential confound of an RBD history, PD patients without RBD during the assessment but with an RBD history were also excluded from the study. Healthy controls with current RBD symptoms or a RBD history were excluded from the current study. In addition, PD+pRBD were interviewed, along with their bed partners, using a structured questionnaire to compare patients during RBD versus wakefulness for quality of movements (speed, smoothness and strength), facial expression and speech (volume of the voice, articulation and intelligibility)^52–54^. They were asked to score each item as ‘much better than awake’, ‘better than awake’, ‘similar to awake’, ‘worse than awake’ or ‘do not know’. To minimize the effects of medication on the results, all clinical measurements and MRI data in all patients were assessed or acquired while they were “off” medication (i.e., testing after overnight withdrawal of antiparkinsonian medication).

### MRI acquisition and preprocessing

Imaging was carried out in a 3 T MR scanner (Skyra system; Siemens Magnetom scanner, Germany) with a standard 12-channel head coil. Participants were instructed to keep their head still, and eyes closed in the scanner and not to think about anything in particular or fall asleep. Tight but comfortable, foam padding was used to restrict head motion. High-resolution anatomic images were acquired with a 3D T1-weighted magnetization prepared rapid acquisition gradient echo (MPRAGE) scan (TR = 2530 ms, TE = 2.98 ms, slice thickness = 1.0 mm, 192 sagittal slices, field of view (FOV) = 256 mm). By contrast, resting-state fMRI data were acquired using a standard gradient-echo echo-planar sequence (TR = 2000 ms, TE = 30 ms, slice thickness = 3 mm, 35 axial slices, 176 time points, Flip angle = 90°, FOV = 220 mm, matrix size = 64 × 64).

Brain imaging data were preprocessed using DPABI software^55^ (http://rfmri.org/ dpabi, Version 6.1) running under MATLAB (R2018b) (The Math-Works Inc., Natick, MA, USA). Structural images were segmented using the unified segmentation model into gray matter (GM), white matter (WM), and cerebrospinal fluid (CSF) based on tissue probability maps in Montreal Neurological Institute (MNI) space. VBM analysis of GM was conducted after being normalized and smoothed using a Gaussian filter kernel with 4 mm full width at half maximum (FWHM).

The preprocessing steps of rs-fMRI data were carried out as follows: (1) the first 10 time points were discarded to allow for signal stabilization; (2) slice acquisition timing discrepancies and head motion were corrected in the remaining functional images; (3) linear trend, Friston 24 head-motion parameters, the white matter signal and cerebrospinal fluid signal were regressed out from the functional signal as nuisance covariates; (4) derived functional images were coregistered with the corresponding structural images which were segmented and normalized to MNI space using the Diffeomorphic Anatomic Registration with the Exponentiated Lie (DARTEL); (5) the functional images were then normalized to MNI space with warped parameters, and resampled to 3 mm cubic voxels; (6) temporal bandpass filtering (0.01-0.1 Hz) on all functional images were then performed. The outputs were visually inspected for accuracy, and manual edits and reprocessing were performed if needed.

Participants with poor-quality images, including bad quality spatial normalization, inadequate brain coverage ( < 90% group brain mask coverage), and maximum head motion larger than 2 mm in displacement or 2^◦^ rotation, as well as mean frame-wise displacement (FD, derived from Jenkinson’s relative root mean square algorithm) larger than 0.2 mm, were excluded in this study. Overall, 18 subjects (6 controls, 7 PD+pRBD and 5 PD-pRBD) with poor coverage and 14 subjects (4 controls, 5 PD+pRBD and 5 PD-pRBD) with excessive head motion were excluded, as well as 4 subjects (1 control, 2 PD+pRBD and 1 PD-pRBD) with large FD. Finally, 36 PD+pRBD, 57 PD-pRBD, and 71 healthy controls were retained for final analysis (Supplementary Fig. 1).

### Brain network construction

For network analyses, the whole brain was first parcellated into 160 cortical and subcortical functional ROIs (or nodes) using the Dosenbach atlas^56^. Each node was a sphere centered on the atlas coordinates, with a radius of 5 mm. Then, the neural signal of each node was derived by averaging the preprocessed blood oxygen level-dependent (BOLD) signals of all voxels within the sphere. To derive the connectivity matrix of the brain, FC for any pair of two ROIs was computed as the Pearson’s correlation coefficient of the BOLD signals, which was then transformed to z values using Fisher’s r-to-z formula. For each subject, the weighted topological parameters of the correlation matrices over a wide range of network edge density thresholds (10% ≤ density ≤ 34%, step of 1%, similar to a previous study^57^) were further calculated. The network was constructed using DPABINet (version 1.1) implemented in DPABI^55^, version 6.1(http://rfmri.org/ dpabi).

### Network analysis

The global and regional topological properties of brain graphs were calculated at each density threshold. At the global level, we examined global efficiency (E_glob_), local efficiency (E_loc_), assortativity coefficient, and modularity. The path length (L_p_) and clustering coefficient (C_p_) were used in validation analysis since they generally reflect the same information as E_glob_ and E_loc_^58,59^. E_glob_ (Eq. (1)) was defined as how efficiently the whole network exchanges information, computed as follows:

1 $${E}_{glob}^{w}=\frac{1}{n}\sum _{i\in N}{E}_{i}^{w}=\frac{1}{n}\sum _{i\in N}\frac{{\sum }_{j\in N,j\ne i}{\left({d}_{ij}^{w}\right)}^{-1}}{n-1}$$

where E_i_ indicates node i’s weighted efficiency and d_ij_^w^ indicates the shortest weighted path length between nodes i and j.

The efficiency of information exchange within local subnetworks was quantified as E_loc_ (Eq. (2)). It was calculated as follows:

2 $${E}_{loc}^{w}=\frac{1}{2}\sum _{i\in N}{E}_{loc,i}^{w}=\frac{1}{2}\sum _{i\in N}\frac{{\sum }_{j,h\in N,j\ne i}{\left({w}_{ij}{w}_{ih}{\left[{d}_{jh}^{w}({N}_{i})\right]}^{-1}\right)}^{1/3}}{{k}_{i}({k}_{i}-1)}$$

where E_loc,i_^w^ indicates the weighted local efficiency of node i. The connection weights between nodes i and j were denoted by w_ij_, and d_jh_^w^(N_i_) indicates the weighted length of the shortest path between j and h, composed exclusively of the neighbors of i.

Assortativity coefficient (Eq. (3)) was the tendency of nodes to link with those nodes with similar number of edges and computed as:

3 $${r}^{w}=\frac{{l}^{-1}{{\sum }_{(i,j)\in L}{w}_{ij}{k}_{i}^{w}{k}_{j}^{w}-\left[{l}^{-1}{\sum }_{(i,j)\in L}\frac{1}{2}{w}_{ij}\left({k}_{i}^{w}+{k}_{j}^{w}\right)\right]}^{2}}{{l}^{-1}{\sum }_{(i,j)\in L}\frac{1}{2}{w}_{ij}\left({\left({k}_{i}^{w}\right)}^{2}+{\left({k}_{j}^{w}\right)}^{2}\right)-{\left[{l}^{-1}{\sum }_{(i,j)\in L}\frac{1}{2}{w}_{ij}\left({k}_{i}^{w}+{k}_{j}^{w}\right)\right]}^{2}}$$

Where *L* was the set of all links in the network, and *l* was the total weight of all links in the network. w_ij_ indicates the connection weights associated with links (i, j).

Modularity (Eq. (4)) was the extent to which a graph could be segregated into densely intraconnected but sparsely interconnected modules. It was calculated as follows:

4 $${Q}^{w}=\frac{1}{{l}^{w}}\mathop{\sum} \limits_{i,j\in N}\left[{w}_{ij}-\frac{{k}_{i}^{w}{k}_{j}^{w}}{{l}^{w}}\right]{\delta }_{{m}_{i},{m}_{j}}$$

where m_i_ was the module containing node i, and δmi,mj = 1 if mi = mj, and 0 otherwise.

L_P_ (Eq. (5)) was equivalent to the inverse of E_glob_. It can be described as:

5 $${L}_{p}^{w}=\frac{1}{n}\sum _{i\in N}{L}_{i}^{w}=\frac{1}{n}\sum _{i\in N}\frac{{\sum }_{j\in N,j\ne i}{d}_{ij}^{w}}{n-1}$$

where L_i_^w^ indicates the mean weighted distance between node i and all the rest nodes. d_ij_^w^ indicates the shortest weighted length of the path between i and j.

C_p_ (Eq. (6)) generally reflects similar information as E_loc_ and is computed as:

6 $${C}_{p}^{w}=\frac{1}{n}\sum _{i\in N}{C}_{i}=\frac{1}{n}\sum _{i\in N}\frac{2{t}_{i}^{w}}{{k}_{i}({k}_{i}-1)}$$

where C_i_^w^ was the node i’s weighted clustering coefficient (C_i_^w^ = 0 if k_i_ < 2), k_i_ was the node i’s degree, and t_i_^w^ was the geometric mean of triangles in the vicinity of i.

At the regional level, we examined the degree, betweenness and node efficiency for each node. Degree (Eq. (7)) was defined as the sum of links’ weights connected to a node and computed as:

7 $${k}_{i}^{w}=\sum _{j\in N}{w}_{ij}$$

Betweenness (Eq. (8)) was the fraction of all shortest paths in the graph that pass through a particular node, which was computed as:

8 $${b}_{i}=\frac{1}{(n-1)(n-2)}\sum _{\begin{array}{c}h,j\in N\\ h\ne j,h\ne i,i\ne j\end{array}}\frac{{\rho }_{hj}(i)}{{\rho }_{hj}}$$

where ρ_hj_ was the number of shortest paths between node h and j, and ρ_hj_ (i) stood for the number of shortest paths between node h and j that passed through i.

Nodal efficiency (Eq. (9)) was a measure of how efficiently the information can flow through a particular node to reach other nodes in the network and computed as:

9 $${E}_{i}^{w}=\frac{1}{n-1}\sum _{j\in N,j\ne i}\frac{1}{{d}_{ij}^{w}}$$

For each network metric, the area under the curve (AUC) across the density range was calculated^60^.

To facilitate data interpretation, we arranged the nodes and reported the brain networks based on the networks defined by Yeo et al. ^61^. The Yeo atlas divided the human cortex into seven networks. The limbic network from Yeo et al. was not included in the present study because none of the 160 Dosenbach ROIs were located within this network. Instead, we defined subcortical ROIs as the “subcortical network” and also included the “cerebellar network” from the Dosenbach ROIs in our eight-networks model. The eight networks were: the cerebellar network (CBN, 18 ROIs located in the cerebellum), default mode network (DMN, 33 ROIs located in the inferior parietal lobule, posterior cingulate cortex, lateral temporal cortex, and ventral and medial prefrontal cortex), frontoparietal network (FPN, 21 ROIs located in the superior parietal lobule, precuneus, lateral frontal cortex, and dorsal cingulate cortex), subcortical network (SCN, 7 ROIs located in the putamen and thalamus), ventral attention network (VAN,16 ROIs located in the supramarginal gyrus, insula, middle frontal gyrus, supplementary motor area), dorsal attention network (DAN, 14 ROIs located in the temporo-occipital cortex, angular gyrus, superior parietal lobule, and premotor cortex), somatosensory-motor network (SMN, 29 ROIs located in the precentral and postcentral gyrus and auditory cortex), and visual network (VN, 22 ROIs located in the occipital lobe and posterior fusiform gyrus).

### Statistical analysis

Group differences in the demographic and clinical characteristics of the study participants were examined using the Chi-square test for categorical variables and the two-sample Student *t* test and/or analysis of variance (ANOVA) for continuous variables. The threshold used for statistical significance was set (with Bonferroni correction) at less than 0.05 (SPSS for Windows, Version 21.0, SPSS, Chicago, IL, USA). Statistical analyses of the imaging data were carried out using DPABI software, running under MATLAB.

To determine whether there were significant group differences in the network topological properties, the AUC values of each global network measure and each nodal network measure across 160 nodes were compared using general linear model with age, sex, educational level, and head motion (i.e., mean values of FD) included as nuisance covariates. Multiple comparisons for nodal network properties were corrected for FDR correction. To localize specific pairs of brain regions in which FC was altered in patient-control contrasts, the network-based statistics (NBS; https://www.nitrc.org/projects/nbs) approach was used^21^.

NBS can provide more statistical power than mass-univariate analysis^21^. For the 12720 pairs of ROIs (160 ×159/2), NBS analyses with *t*-tests were conducted to compare FC between group contrasts among PD+pRBD, PD-pRBD and controls, respectively, with sex, age, educational level and head motion were added in the linear model as covariates. The primary threshold was set at P < 0.001 (two tailed) in *t*-test for every edge and permutations with 5000 iterations were employed to generate distributions of suprathreshold edge numbers in the cluster with the most suprathreshold edges. The patient-control contrasts of NBS analysis were assessed with a significance level using a 2-tailed component p value < 0.05

Correlation analyses were performed between the network measures showing between-group differences and clinical variables, including MoCA score, RBDQ-HK score, HAMD score, UPDRS motor score, H&Y stage, disease duration in PD+pRBD as well as PD-pRBD. For MoCA score, RBDQ-HK score, and HAMD score, these correlation analyses were also conducted in healthy controls. To avoid confounding factors, further correlation analyses with age, sex, educational level and head motion as covariates were also performed. The statistical threshold of the correlation analysis was set at p < 0.05 (2-tailed), corrected via FDR correction for multiple comparisons. The estimate of effect size for all two-group comparisons and the correlation analyses were measured by Cohen’s d.

### Validation analysis

To test the robustness of our main findings, we performed validation analyses. For the whole-brain topological metrics, we evaluated different topological parameters with equivalent meanings (i.e., C_p_ and L_p_). With the edge-based FC analysis, beyond NBS analysis, we also validated our FC comparisons by analyzing large-scale within- and between -network FC. Moreover, we also defined the nodes using another functional atlas^62^ (i.e., Craddock’s functional clustering atlas) to construct functional brain networks and further analyses the NBS across different parcellation strategies.

For the large-scale network FC analysis, the average FC (Fisher’s r-to-z transformed Pearson’s correlation between time series of all ROI pairs) within each network was defined as within-network FC for patient–control contrasts. Since we defined eight networks, this resulted in eight within-network averaged FC values, and 28 between-network averaged FC values. The large-scale network FC values were compared between patient-control contrasts with two sample t tests, controlling for age, sex educational level and head motion. FDR corrections were employed to correct for multiple comparisons across eight within-network and 28 between-network FC values (corrected to p < .005).

To test whether our main findings could be replicated, we employed a different atlas and re-ran the NBS analyses. The atlas developed by Craddock et al. (2012) was divided the brain into 200 regions through a functionally clustering method. For 19900 pairs of these regions (200 ×199/2), FC was computed for each participant and each condition, and NBS analyses with *t*-tests were conducted to examine the FC between patients and healthy controls.

In addition, to assess the potential structural changes, VBM analyses were implemented for structural imaging with a 2-tailed significance level of p < 0.05, after correcting via FDR for multiple comparisons. By further controlling the clinical measurements correlated with the network metrics, the potential moderators of the brain network changes in patients were also validated.

### Supplementary information

Supplementary Information

Related Manuscript File

## Data Availability

Clinical and neuroimaging data in the present study is not publicly available as it contains information that could breach research participant privacy/consent, but can be shared by contacting the corresponding authors based on reasonable requests from qualified researchers, within the limitations of the provided informed consent. Every request will be reviewed by the Institutional Review Board of the Xuanwu Hospital of Capital Medical University, China, and the requesting researcher will need to sign a data access agreement after approval.

## Code availability

DPABI (v6.1, http://rfmri.org/ dpabi), MATLAB R2018b (http://www.mathworks.com) and SPM12 (http://www.fil.ion.ucl.ac.uk/spm) in the study are open sources.
