# Supplementary material for: Reorganization of intrinsic functional connectivity in early-stage Parkinson’s disease patients with probable REM sleep behavior disorder
Source: NPJ Parkinsons Dis. 2024 Jan 3;10:5. doi: 10.1038/s41531-023-00617-7 (PMC10764752; doi:10.1038/s41531-023-00617-7)
Supplement: Supplementary file 1 — Supplementary Information [file 41531_2023_617_MOESM1_ESM.pdf]

Supplementary Information

1 The results of movements for patients with PD during RBD

Thirty-two bed partners were able to compare the quality of movements, facial expression, and speech expressions of their co-sleeper during RBD [3] to similar awake behavior. One partner of PD+pRBD said they did not sleep in the same room, the other partner of PD+pRBD said that the room was too dark to evaluate these aspects. Twenty-nie (90.6%) bed partners reported an improvement of at least one component of motor control during RBD. The movements were improved in 81.3% of PD+pRBD including faster (78.1%), stronger (78.1%), or smoother (53.1%). Speech was better in 59.3% of PD+pRBD and was more intelligible (46.9%), louder (50.0%) or better articulated (59.3%). Facial expressions were normalized in 53.1% patients during the RBD. Based on the bed-partner interview, while the patients had asymmetrical parkinsonism when awake, most of the time they used the more disabled arm, hand and leg during the RBD. The improvement of motor function during RBD in patients with PD was observed in this study, although future fMRI study in PD+pRBD with both during RBD and wakefulness states would be needed to elucidate the underlying alteration in brain function for this phenomenon.

2 The results of validation analyses

2.1 The results of large-scale FC analysis

Compared to healthy controls, large-scale network FC demonstrated decreased within-network FC of the SMN, FPN, CBN and DAN, as well as extensively decreased between-network FC (all 28/28 between-networks in PD+pRBD (Fig. 4a and Supplementary Table 3). In contrast, as shown in Fig.4b and Supplementary Table 4, only decreased within-network FC of FPN and no statistically significant between-network FC were found in PD-pRBD compared to healthy controls after FDR correction. For comparison between PD+pRBD and PR-pRBD, none of within-network or between-network FC values differed significantly.

2.2 The results of replicated analyses using atlas developed by Craddock et al.

NBS analyses with t-tests were conducted to compare FC between PD+pRBD, PD-pRBD, and healthy controls after controlling for age, sex, educational level and mean FD. This revealed a significant cluster consisting of 182 ROIs and 1207 edges with decreased FC in patients with PD+pRBD as compared to healthy controls, and a significant cluster consisting of 61 ROIs and 88 edges with decreased FC in patients with PD-pRBD as compared to healthy controls. This result is broadly in line with the finding using the Dosenbach atlas.

3 The results of network analysis between all patients with PD and healthy controls

3.1 Network-based statistics (NBS) analysis results

NBS analysis revealed a significant cluster consisting of 99 ROIs and 217edges with significantly altered FC in PD patients compared to healthy controls (Supplementary Fig. 3c). The suprathreshold edges with decreased FC involved all networks, suggesting extensive disruption of brain networks in pooled PD patients (Supplementary Table 5). More affected edges were connected to ROIs in SMA and FPN, and fewer in the CBN and DAN. All the suprathreshold edges showed decreased FC in PD patients versus HCs. The most significant decreased edge was between the DAN and VAN (T = -5.2279, P =0.0002). The results of brain edge-based FC matrices of healthy controls and PD patients are shown in Supplementary Fig. 3a and Supplementary Fig. 3b, respectively.

3.2 Large-scale network functional connectivity results

In the validation analysis of large-scale network FC, we found that PD patients demonstrated decreased within-network FC of FPN, as well as decreased between-network FC for 8 pairs of networks, including VN-VAN, SCN-DAN, FPN- DAN, DMN-DAN, FPN-VAN, FPN-SCN, DMN-VAN and DMN-FPN, after FDR correction (Supplementary Fig. 4).

Supplementary Figure 1. Flowchart of the study participants in image analyses. Abbreviations: *PD* Parkinson's disease, *HC* healthy controls, *FD* frame-wise displacement.

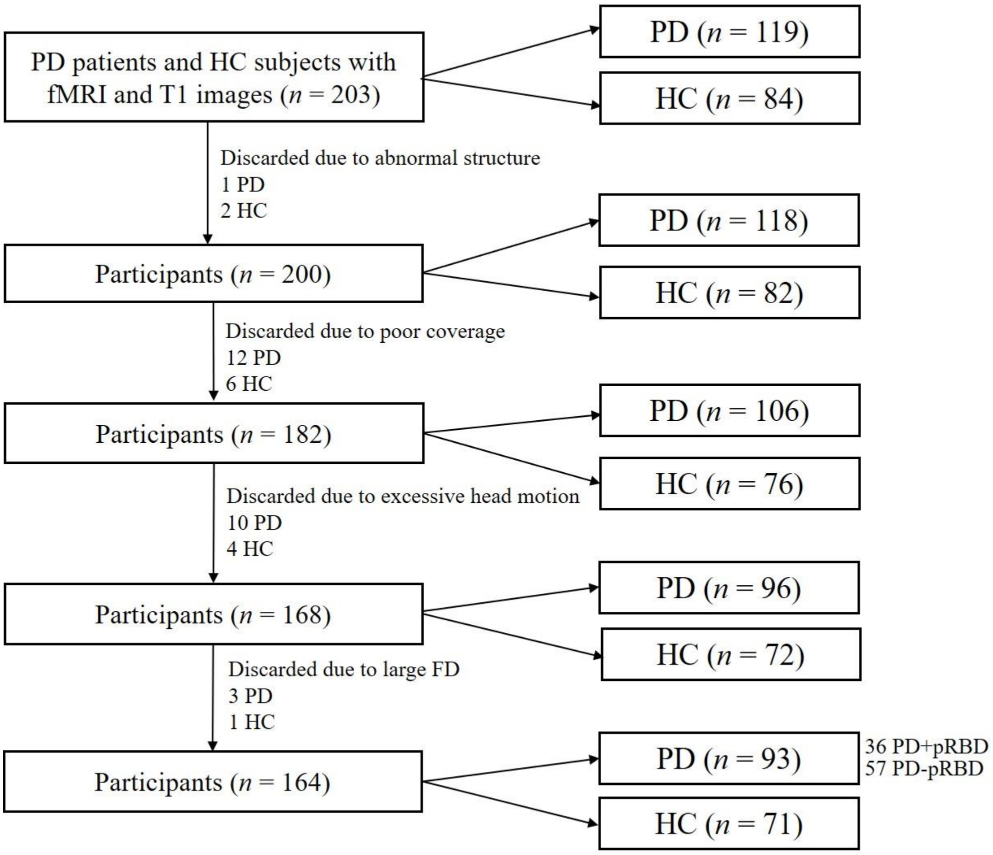

Supplementary Figure 2. The maps of brain edge-based FC matrices of PD+pRBD (n = 36), PD-pRBD (n = 57) and HC (n = 71). (a) FC matrixes of PD+pRBD. (b) FC matrixes of PD-pRBD. (c) FC matrixes of HC. Abbreviations: *FC* functional connectivity, *HC* healthy controls, *PD* Parkinson's disease, *VN* visual network, *SMN* somatosensory network, *DAN* dorsal attention network, *VAN* ventral attention network, *SCN* subcortical network, *FPN* frontoparietal network, *DMN* default mode network, *CBN* cerebellar network.

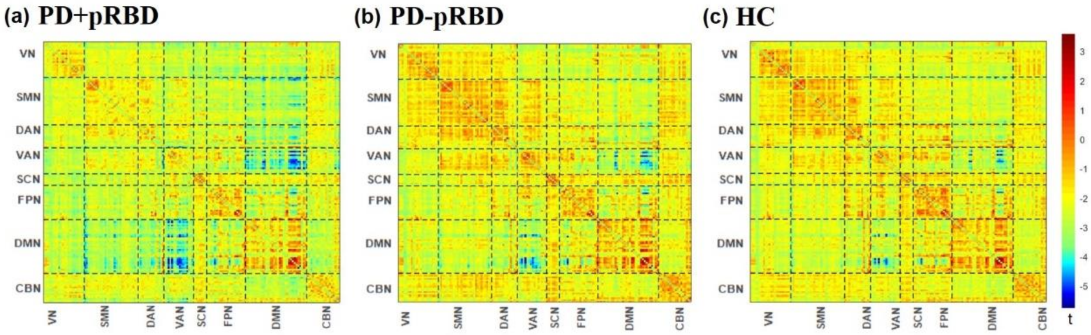

Supplementary Figure 3. The maps of brain edge-based FC matrices of PD (n =93), HC (n = 71) and comparison between PD and HC by NBS analysis. (a) shows FC matrixes of HC. (b) shows FC matrixes of PD. (c) shows brain network view of the group differences between PD and HC by NBS analysis. Abbreviations: *FC* functional connectivity, *HC* healthy controls, *PD* Parkinson's disease, *NBS* Network-Based Statistic, *VN* visual network, *SMN* somatosensory network, *DAN* dorsal attention network, *VAN* ventral attention network, *SCN* subcortical network, *FPN* frontoparietal network, *DMN* default mode network, *CBN* cerebellar network.

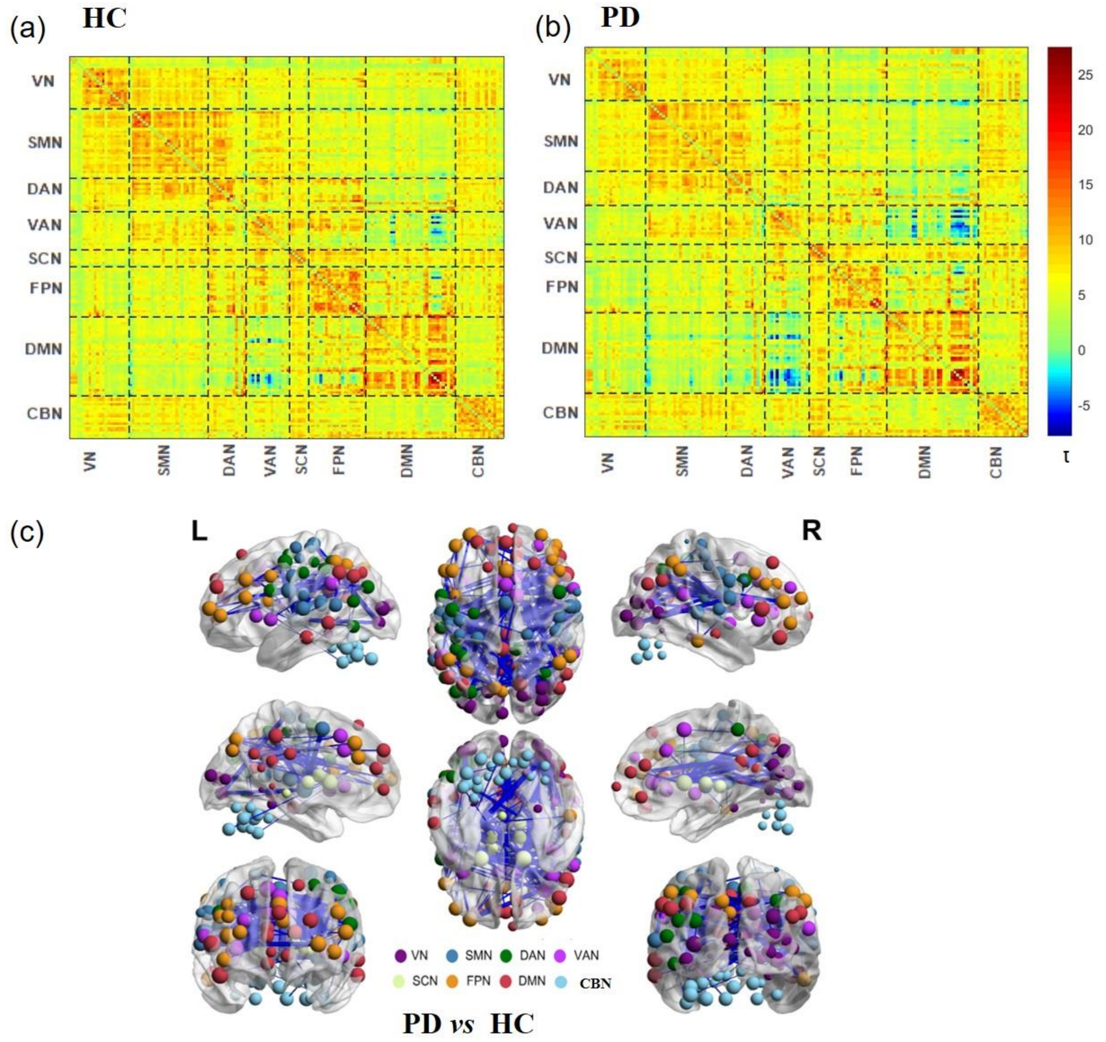

Supplementary Figure 4. The large-scale within- and between-network FC comparisons between PD and HC. The left heatmap shows the T values of two sample T tests on large-scale network FC comparison between PD and HC. The schematic diagram on right shows the network connections with significant FC decrease for the eight networks between PD and HC. Abbreviations: *FC* functional connectivity, *PD* Parkinson's disease, *HC* healthy controls, *VN* visual network, *SMN* somatosensory network, *DAN* dorsal attention network, *VAN* ventral attention network, *SCN* subcortical network, *FPN* frontoparietal network, *DMN* default mode network, *CBN* cerebellar network. * Significant FDR-corrected p < .05 (two-tailed) among eight within network and 28 between-network connections.

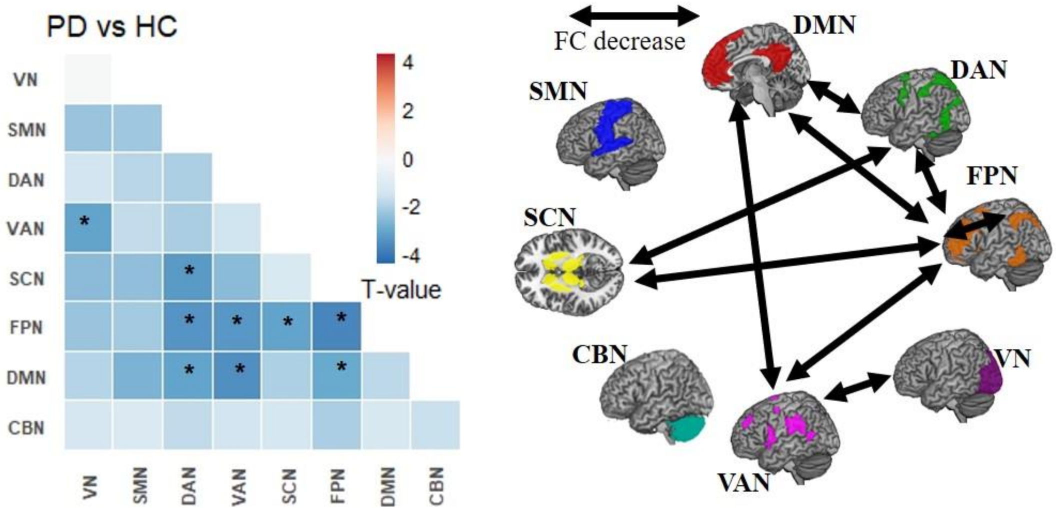

Supplementary Table 1 Number and ratio of significant ROI-wise FC in PD patients with RBD (N =36) compared with healthy controls (N = 71)

Number<br>Percent	VN	SMN	DAN	VAN	SCN	FPN	DMN	CBN
VN	0<br>0%							
SMN	37<br>5.80%	56<br>13.80%						
DAN	3<br>0.97%	18<br>4.43%	4<br>4.40%					
VAN	16<br>4.55%	9<br>1.94%	2<br>0.89%	1<br>0.83%				
SCN	9<br>5.84%	14<br>6.90%	13<br>13.27%	5<br>4.46%	0<br>0%			
FPN	8<br>1.73%	9<br>1.48%	13<br>4.42%	13<br>3.87%	7<br>4.76%	4<br>1.90%		
DMN	8<br>1.10%	43<br>4.49%	19<br>4.11%	25<br>4.73%	2<br>0.87%	6<br>0.87%	4<br>0.76%	
CBN	0<br>0%	9<br>1.72%	1<br>0.40%	7<br>2.43%	0<br>0%	2<br>0.53%	6<br>1.01%	3<br>1.96%

Values in the first line of each cell are the count number of suprathreshold edges belonging to each pair of networks for the significant cluster obtained from the NBS analysis; while values in the second line are the ratio (in percent) of that number to the number of full connections for each pair of networks. The bold values mean the edges / ratios showed increased FC in PD patients with RBD compared with healthy controls, otherwise means the edges / ratios showed decreased FC in PD patients with RBD compared with healthy controls. VN, visual network; SMN, somatosensory network; DAN, dorsal attention network; VAN, ventral attention network; SCN, subcortical network; FPN, frontoparietal network; DMN, default mode network; CBN, cerebellar network.

Supplementary Table 2 Number and ratio of decreased ROI-wise FC in PD patients with RBD (N = 57) compared with healthy controls (N = 71)

Number<br>Percent	VN	SMN	DAN	VAN	SCN	FPN	DMN	CBN
VN	0<br>0%							
SMN	6<br>0.94%	7<br>1.72%						
DAN	0<br>0%	4<br>0.99%	0<br>0%					
VAN	3<br>0.85%	1<br>0.22%	0<br>0%	1<br>0.83%				
SCN	1<br>0.65%	1<br>0.49%	2<br>2.04%	0<br>0%	0<br>0%			
FPN	1<br>0.22%	1<br>0.16%	0<br>0%	15<br>4.46%	2<br>1.36%	6<br>2.86%		
DMN	0<br>0%	4<br>0.42%	4<br>0.87%	4<br>0.76%	1<br>0.43%	9<br>1.30%	1<br>0.19%	
CBN	0<br>0%	1<br>0.19%	0<br>0%	0<br>0%	0<br>0%	3<br>0.79%	0<br>0%	0<br>0%

Values in the first line of each cell are the count number of suprathreshold edges belonging to each pair of networks for the significant cluster obtained from the NBS analysis; while values in the second line are the ratio (in percent) of that number to the number of full connections for each pair of networks. VN, visual network; SMN, somatosensory network; DAN, dorsal attention network; VAN, ventral attention network; SCN, subcortical network; FPN, frontoparietal network; DMN, default mode network; CBN, cerebellar network.

Supplementary Table 3 Large-scale network FC comparisons between PD patients with RBD (n=36) and healthy controls (n=71)

	VN	SMN	DAN	VAN	SCN	FPN	DMN	CBN
VN	-0.91							
SMN	-3.02	-3.78						
DAN	-2.28	-2.91	-2.76					
VAN	-3.43	-2.89	-2.86	-2.72				
SCN	-2.79	-2.98	-3.29	-3.20	-2.05			
FPN	-2.64	-2.48	-3.32	-3.44	-2.70	-3.04		
DMN	-2.47	-3.26	-3.35	-3.58	-2.25	-2.64	-1.62	
CBN	-2.26	-2.47	-2.60	-2.63	-2.51	-2.56	-2.28	-3.08

VN, visual network; SMN, somatosensory network; DAN, dorsal attention network; VAN, ventral attention network; SCN, subcortical network; FPN, frontoparietal network; DMN, default mode network; CBN, cerebellar network.

Supplementary Table 4 Large-scale network FC comparisons between PD patients without RBD (n=57) and healthy controls (n=71)

	VN	SMN	DAN	VAN	SCN	FPN	DMN	CBN
VN	0.07							
SMN	-1.48	-0.86						
DAN	-0.89	-0.94	-1.22					
VAN	-2.04	-0.72	-1.33	-1.24				
SCN	-1.58	-1.28	-2.08	-1.71	-0.63			
FPN	-1.74	-1.39	-2.44	-2.74	-2.45	-3.32		
DMN	-1.31	-1.52	-1.90	-2.16	-1.08	-2.16	-1.11	
CBN	-0.71	-0.46	-1.20	-0.99	-0.51	-1.72	-0.57	-0.58

VN, visual network; SMN, somatosensory network; DAN, dorsal attention network; VAN, ventral attention network; SCN, subcortical network; FPN, frontoparietal network; DMN, default mode network; CBN, cerebellar network.

Supplementary Table 5 Number and ratio of significant ROI-wise FC in PD patients (N =93) compared with healthy controls (N = 71)

Number<br>Percent	VN	SMN	DAN	VAN	SCN	FPN	DMN	CBN
VN	0<br>0%							
SMN	27<br>4.23%	17<br>4.19%						
DAN	0<br>0%	10<br>2.46%	2<br>2.20%					
VAN	7<br>1.99%	4<br>0.86%	4<br>1.79%	1<br>0.83%				
SCN	6<br>3.90%	6<br>2.96%	13<br>13.27%	4<br>3.57%	0<br>0%			
FPN	3<br>0.65%	8<br>1.31%	12<br>4.08%	16<br>4.76%	8<br>5.44%	4<br>1.90%		
DMN	6<br>0.83%	13<br>1.36%	7<br>1.52%	15<br>2.84%	5<br>2.16%	7<br>1.01%	4<br>0.76%	
CBN	2<br>0.51%	1<br>0.19%	0<br>0%	0<br>0%	0<br>0%	2<br>0.53%	3<br>0.51%	0<br>0%

Values in the first line of each cell are the count number of suprathreshold edges belonging to each pair of networks for the significant cluster obtained from the NBS analysis; while values in the second line are the ratio (in percent) of that number to the number of full connections for each pair of networks. The bold values mean the edges / ratios showed increased FC in PD patients compared with healthy controls, otherwise means the edges / ratios showed decreased FC in PD patients compared with healthy controls. *FC* functional connectivity, *PD* Parkinson’s disease, *NBS* Network-Based Statistic, *VN* visual network, *SMN* somatosensory network, *DAN* dorsal attention network, *VAN* ventral attention network, *SCN* subcortical network, *FPN* frontoparietal network, *DMN* default mode network, *CBN* cerebellar network.

Supplementary Table 6 Functional MRI studies in PD patients with RBD

Authors	Participants	Sample size	Diagnosis of RBD	Analyses approaches
Gallea et al. (2017)	PD+RBD<br>PD-RBD<br>Healthy controls	22<br>14<br>25	PSG	Seed-based functional connectivity
Li et al. （2017）	PD+RBD<br>PD-RBD<br>Healthy controls	18<br>16<br>19	PSG	Amplitude of low frequency fluctuations
Li et al. (2020)	PD+RBD<br>PD-RBD<br>Healthy controls	30<br>62<br>20	Questionnaire	graph theory approaches.
Liu et al. (2021)	PD+RBD<br>PD-RBD<br>Healthy controls	19<br>19<br>20	Questionnaire	Regional homogeneity (ReHo), functional connectivity
Oltra et al. (2021)	PD+RBD<br>PD-RBD<br>Healthy controls	27<br>31<br>30	Questionnaire	whole-brain network-based statistics and graph-theoretical approaches
Jiang et al. (2021)	PD+RBD<br>PD-RBD<br>Healthy controls	24<br>26<br>26	PSG	Seed-based functional connectivity
Jia et al. (2021)	PD+RBD<br>PD-RBD<br>Healthy controls	18<br>28<br>22	Questionnaire	Seed-to-voxel functional connectivity analysis
Gan et al. (2021)	PD+RBD<br>PD-RBD<br>Healthy controls	45<br>81<br>37	Questionnaire	independent component analysis, sliding window approach, k-means clustering methods
