# Supplementary material for: Reorganization of intrinsic functional connectivity in early-stage Parkinson’s disease patients with probable REM sleep behavior disorder
Source: NPJ Parkinsons Dis. 2024 Jan 3;10:5. doi: 10.1038/s41531-023-00617-7 (PMC10764752; doi:10.1038/s41531-023-00617-7)
Supplement: Supplementary file 2 — Related Manuscript File [file 41531_2023_617_MOESM2_ESM.pdf]

nature portfolio

Corresponding author(s): Tao Wu, Piu Chan

Last updated by author(s): Dec 6, 2023

Reporting Summary

Nature Portfolio wishes to improve the reproducibility of the work that we publish. This form provides structure for consistency and transparency in reporting. For further information on Nature Portfolio policies, see our Editorial Policies and the Editorial Policy Checklist.

Statistics

For all statistical analyses, confirm that the following items are present in the figure legend, table legend, main text, or Methods section.

n/a	Confirmed	
☐	☒	The exact sample size (*n*) for each experimental group/condition, given as a discrete number and unit of measurement
☐	☒	A statement on whether measurements were taken from distinct samples or whether the same sample was measured repeatedly
☐	☒	The statistical test(s) used AND whether they are one- or two-sided<br>*Only common tests should be described solely by name; describe more complex techniques in the Methods section.*
☐	☒	A description of all covariates tested
☐	☒	A description of any assumptions or corrections, such as tests of normality and adjustment for multiple comparisons
☐	☒	A full description of the statistical parameters including central tendency (e.g. means) or other basic estimates (e.g. regression coefficient) AND variation (e.g. standard deviation) or associated estimates of uncertainty (e.g. confidence intervals)
☐	☒	For null hypothesis testing, the test statistic (e.g. *F*, *t*, *r*) with confidence intervals, effect sizes, degrees of freedom and *P* value noted<br>*Give P values as exact values whenever suitable.*
☒	☐	For Bayesian analysis, information on the choice of priors and Markov chain Monte Carlo settings
☐	☒	For hierarchical and complex designs, identification of the appropriate level for tests and full reporting of outcomes
☐	☒	Estimates of effect sizes (e.g. Cohen's *d*, Pearson's *r*), indicating how they were calculated

Our web collection on statistics for biologists contains articles on many of the points above.

Software and code

Policy information about availability of computer code

Data collection	No code and software used for the data collection
Data analysis	DPABI software(http://rfmri.org/ dpabi, Version 6.1)

For manuscripts utilizing custom algorithms or software that are central to the research but not yet described in published literature, software must be made available to editors and reviewers. We strongly encourage code deposition in a community repository (e.g. GitHub). See the Nature Portfolio guidelines for submitting code & software for further information.

Data

Policy information about availability of data

All manuscripts must include a data availability statement. This statement should provide the following information, where applicable:

- Accession codes, unique identifiers, or web links for publicly available datasets
- A description of any restrictions on data availability
- For clinical datasets or third party data, please ensure that the statement adheres to our policy

The data that support the findings of this study are available from the corresponding authors upon reasonable request.

Research involving human participants, their data, or biological material

Policy information about studies with human participants or human data. See also policy information about sex, gender (identity/presentation), and sexual orientation and race, ethnicity and racism.

Reporting on sex and gender	The term 'sex' was used to avoid confusing. Sex was considered in study design and the ratios between male and female for different study groups were compared. No significant difference for sex were observed between different study groups (p =0.832). The data analyses were based on the anonymous samples. In addition, the data analyses with sex as covariant were also performed in our study. Therefore, the findings were applied to both male and female.
Reporting on race, ethnicity, or other socially relevant groupings	All participants in this study were Chinese Han ethnicity. This information were provided by the self-report of the participants.
Population characteristics	A total of 203 ubjects, including 119 patients with Parkinson's disease(PD) and 84matched healthy controls, participated in this study. In total, 93 PD patients and 71 healthy controls were included in the final sample after implementing quality control measures. PD patients and healthy controls were matched with respect to age, sex and educational level.
Recruitment	The participants were recruited at no biases. The ages of subjects were from 40 to 80. Patients with PD were recruited from the Parkinson and Movement Disorder Center at the Xuanwu Hospital of Capital Medical University in Beijing. Clinically established diagnoses of idiopathic PD were made based on the Movement Disorder Society (MDS) clinical diagnostic criteria. Patients with a positive family history of PD, secondary parkinsonism, or other forms of atypical parkinsonism were excluded. Age and sex gender-matched healthy controls were recruited from the Beijing Longitudinal Study on Aging community cohort.
Ethics oversight	This study was conducted in accordance with the Declaration of Helsinki and approved by the Ethic Review Committee of the Xuanwu Hospital of Capital Medical University. All participants gave written informed consent before inclusion in this study.

Note that full information on the approval of the study protocol must also be provided in the manuscript.

Field-specific reporting

Please select the one below that is the best fit for your research. If you are not sure, read the appropriate sections before making your selection.

☒ Life sciences ☐ Behavioural & social sciences ☐ Ecological, evolutionary & environmental sciences

For a reference copy of the document with all sections, see nature.com/documents/nr-reporting-summary-flat.pdf

Life sciences study design

All studies must disclose on these points even when the disclosure is negative.

Sample size	Sample size: patient group = 119, control group = 84, the sample size for final neuroimaging analysis: patient group = 93 (36 PD with RBD, 57 PD without RBD), control group =71. For the neuroimaging analysis, no statistical method was used to determine the sample size. Sample size was chosen based on previous experience and standards in the field. No less than 20 subjects per group were suggested in this field. We note the current study is almost among the largest sample size of functional MRI studies in PD with RBD to date.
Data exclusions	Participants with poor-quality images, including bad quality spatial normalization, inadequate brain coverage (<90% group brain mask coverage), and maximum head motion larger than 2 mm in displacement or 2◦ rotation, as well as mean frame-wise displacement (FD, derived from Jenkinson's relative root mean square algorithm) larger than 0.2mm, were excluded in this study. Overall, 18 subjects (6 controls, 7 PD+pRBD and 5 PD-pRBD) with poor coverage and 14 subjects (4 controls, 5 PD+pRBD and 5 PD-pRBD) with excessive head motion were excluded, as well as 4 subjects (1 control, 2 PD+pRBD and 1 PD-pRBD) with large FD. The exclusion criteria were pre-established.
Replication	We performed validation analyses to test the robustness of our main findings. All attempts at replication were successful and gave similar results.
Randomization	We don't have related issues. It is observation study and the imaging data was collected for each subject.
Blinding	The persons performed the imaging data preprocessing were unaware of the sample identity. The persons performed the imaging data analyses were blinded to the group allocation.

Reporting for specific materials, systems and methods

We require information from authors about some types of materials, experimental systems and methods used in many studies. Here, indicate whether each material, system or method listed is relevant to your study. If you are not sure if a list item applies to your research, read the appropriate section before selecting a response.

Materials & experimental systems

n/a	Involved in the study
☒	☐ Antibodies
☒	☐ Eukaryotic cell lines
☒	☐ Palaeontology and archaeology
☒	☐ Animals and other organisms
☐	☒ Clinical data
☒	☐ Dual use research of concern
☒	☐ Plants

Methods

n/a	Involved in the study
☒	☐ ChIP-seq
☒	☐ Flow cytometry
☐	☒ MRI-based neuroimaging

Clinical data

Policy information about clinical studies

All manuscripts should comply with the ICMJE guidelines for publication of clinical research and a completed CONSORT checklist must be included with all submissions.

Clinical trial registration: We don't have related issues.

Study protocol: We don't have related issues.

Data collection: Patients with PD were recruited from the Parkinson and Movement Disorder Center at the Xuanwu Hospital of Capital Medical University in Beijing. Healthy controls were recruited from the Beijing Longitudinal Study on Aging community cohort.

Outcomes: We don't have related issues.

Magnetic resonance imaging

Experimental design

Design type: Resting state

Design specifications: For each subject in the experiment, the imaging scanning time includes six minutes structural scanning and 8 minutes functional scanning.

Behavioral performance measures: We don't have related issues.

Acquisition

Imaging type(s): Functional, structural

Field strength: 3 Tesla

Sequence & imaging parameters: For 3D T1-weighted magnetization prepared rapid acquisition gradient echo (MPRAGE) scan: TR=2530ms, TE=2.98 ms, slice thickness=1.0mm, 192 sagittal slices, field of view (FOV)=256mm;
For resting-state fMRI data were acquired using a standard gradient-echo echo-planar sequence: TR=2000ms, TE=30 ms, slice thickness=3 mm, 35 axial slices, 176 time points, Flip angle=90°, FOV=220mm, matrix size=64×64.

Area of acquisition: A whole brain scan was used

Diffusion MRI: ☐ Used ☒ Not used

Preprocessing

Preprocessing software: Brain imaging data were preprocessed with the Data Processing Assistant for Resting-State fMRI (DPARSF; http://rfmri.org/DPARSF), which is based on MATLAB (R2018b) (The Math-Works Inc., Natick, MA, USA), SPM12 (http://www.fil.ion.ucl.ac.uk/spm), and DPABI software (http://rfmri.org/ dpabi, Version 6.1). Structural images were segmented using the unified segmentation model into gray matter (GM), white matter (WM), and cerebrospinal fluid (CSF) based on tissue probability maps in Montreal Neurological Institute (MNI) space. Voxel_based morphometry (VBM) analysis of GM was conducted after being normalized and smoothed using a Gaussian filter kernel with 4mm full width at half maximum (FWHM).

Normalization: The Diffeomorphic Anatomical Registration Through Exponentiated Lie algebra (DARTEL) tool was used to normalize functional images to Montreal Neurological Institute (MNI) space.

Normalization template: The MNI152 template was used for spatial normalization.

Noise and artifact removal: The first 10 functional images were removed to allow for signal stabilization. The nuisance signals were regressed out,

Noise and artifact removal	including white matter signal, cerebrospinal fluid signal, linear trend, and the signal associated with the 24 Friston head-motion parameters.
Volume censoring	Dpabi software was used and we have discarded time points which have frame-wise displacement > 0.2 mm

Statistical modeling & inference

Model type and settings	For network-based statistic (NBS) analyses, in the first level, the primary threshold was set at $P < 0.001$ (two tailed) in t-test for every edge and permutations with 5000 iterations were employed to generate distributions of suprathreshold edge numbers in the cluster with the most suprathreshold edges. In the second level, the patient-control contrasts of NBS analysis were assessed with a significance level using a 2-tailed component p value < 0.05.
Effect(s) tested	No task or stimulus conditions were used in this study. For two-group comparisons and the correlation analyses, we calculated the Cohen's d to estimate the effect size.

Specify type of analysis: ☐ Whole brain ☐ ROI-based ☒ Both

Anatomical location(s)	For the structural data, the VBM analysis was used. While for the functional imaging, the graph-theoretical approaches and network-based statistical analyses were used. The Dosenbach atlas was used in functional connectivity analyses and Craddock's functional clustering atlas was used in the validation analyses.
Statistic type for inference (See Eklund et al. 2016)	For the structural data, voxel-wise analyses were used (parameters: individual voxel p <0.001, FDR corrected significance cluster level of p < 0.05, two tailed).<br>For the network-based statistical (NBS) analyses, the primary threshold was set at $P < 0.001$ (two tailed) in T-test for every edge and permutations with 5000 iterations were employed to generate distributions of suprathreshold edge numbers in the cluster with the most suprathreshold edges. The patient-control contrasts of NBS analysis were tests assessed with a significance level at using a 2-tailed component p value < 0.05.
Correction	All the multiple comparisons in this study were corrected. The edge-based functional connectivities were analyzed by NBS with permutation. While the other image data analyses results were corrected via FDR correction for multiple comparisons.

Models & analysis

n/a	Involved in the study
☐	☒ Functional and/or effective connectivity
☐	☒ Graph analysis
☒	☐ Multivariate modeling or predictive analysis

Functional and/or effective connectivity	Functional connectivity for any pair of two ROIs was computed as the Pearson's correlation coefficient of the BOLD signals.
Graph analysis	For each subject, the weighted topological parameters of the correlation matrices over a wide range of network edge density thresholds (10% ≤ density ≤ 34%, step of 1%) were calculated. The global and regional topological properties of brain graphs were calculated at each density threshold. At the global level, we examined global efficiency (Eglob) and local efficiency (Eloc). Path length (Lp) and clustering coefficient (Cp) were used in validation analysis as they generally reflect the same information. At the regional level, we examined the degree, betweenness and node efficiency for each node. For each network metric, the area under the curve (AUC) across the density range was calculated.
